# E-health interventions in postpartum stress: needs assessment and developmental steps towards online interventions

**DOI:** 10.1192/j.eurpsy.2025.90

**Published:** 2025-08-26

**Authors:** K. Koelkebeck, A. Bäuerle

**Affiliations:** 1 Department of Psychiatry and Psychotherapy, LVR-University Hospital Essen, Essen; 2 Protestant Hospital of the Bethel Foundation, University Hospital for Psychiatry and Psychotherapy, Bielefeld; 3 Department of Psychosomatic Medicine and Psychotherapy, LVR-University Hospital Essen, Essen, Germany

## Abstract

**Abstract:**

Postpartum stress and mental disorders have a high prevalence in the population. Postpartum depressed states, for example, potentially threaten care of and bonding with the children. Although mothers face specific needs, specialized treatment options are scarce. Online programs to inform on and treat postpartum stress and depression are, to present, not widely available, but have the potential to overcome some of the obstacles of postpartum women finding treatment.

To identify needs of this specific group, we conducted an online survey on women after childbirth, asking for acceptance of e-health programs, sociodemographic, medical and psychometric data.

In a large, anonymized online survey, 453 women have participated. We investigated 1) the acceptance of tailored e-mental health programs according to the UTAUT model in the respective women and 2) characteristics and needs of the specific populations. Based on our findings, we developed an online tool for stress reduction after child-birth based on relevant topics indicated by and data from the literature on specific needs of post-partum women.

In this talk, motivation for this project, research results and ongoing research will be highlighted and discussed.

**Disclosure of Interest:**

None Declared

